# 297. Prevalence and Genomic Analysis of Hypermucoviscous *Klebsiella pneumoniae* in a Single Center in Japan: Insights into Virulence and Potential Clinical Implications

**DOI:** 10.1093/ofid/ofad500.369

**Published:** 2023-11-27

**Authors:** Naoya Nishiyama, Wakako Arakaki, Kohei Uechi, Daisuke Utsumi, Yukuto Sato, Masashi Nakamatsu, Takeshi Kinjo, Kazuko Yamamoto

**Affiliations:** University of the Ryukyus Graduate School of Medicine, Nishihara, Okinawa, Japan; University of the Ryukyus Graduate School of Medicine, Nishihara, Okinawa, Japan; University of the Ryukyus Hospital, Nishihara, Okinawa, Japan; Graduate School of Medicine, University of the Ryukyus, Nishihara, Okinawa, Japan; University of the Ryukyus, Nishihara, Okinawa, Japan; University of the Ryukyus Graduate School of Medicine, Nishihara, Okinawa, Japan; University of the Ryukyus Graduate School of Medicine, Nishihara, Okinawa, Japan; University of the Ryukyus Graduate School of Medicine, Nishihara, Okinawa, Japan

## Abstract

**Background:**

Hypervirulent *Klebsiella pneumoniae* (hvKp) is associated with severe clinical outcomes, including liver abscesses and disseminated infections (Fig 1). The hypermucoviscosity phenotype (HMV), confirmed by the String test, has been linked to hvKP. This study aimed to investigate the prevalence and genomic characteristics of HMV-Kp isolates in patients with *K. pneumoniae* bacteremia in a single medical center in Japan.

**Figure 1.**
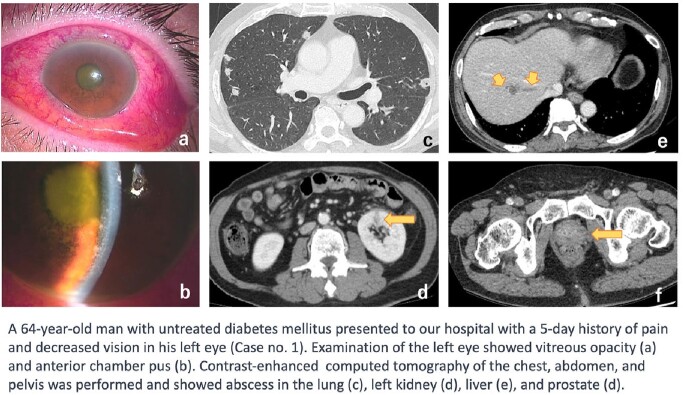
The case of endophthalmitis and multiple abscesses by hypermucovisucosity phenotype K. pneumoniae

**Methods:**

We conducted a retrospective study at University of the Ryukyus Hospital between January 2021 and November 2022, extracting patients with HMV-Kp bacteremia. *K. pneumoniae* isolates were subjected to String test (Fig 2), antimicrobial susceptibility testing, and genomic analysis. Whole-genome sequencing and genomic analysis were conducted to identify multilocus sequence type (MLST), K capsule serotype, virulence genes, and antimicrobial resistance genes.

**Figure 2.**
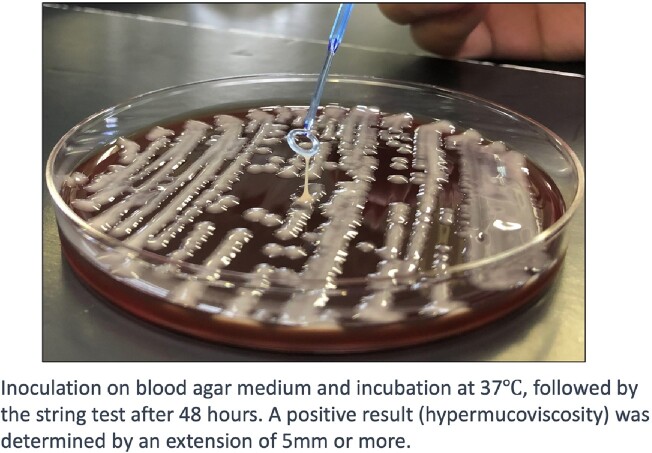
String test

**Results:**

During the study period, 29 cases of *K. pneumoniae* bacteremia were identified, with six cases of HMV-Kp bacteremia. Five of these cases were male patients and had organ abscess formation (Table 1). The results of the genomic analysis are shown in Table 2. All six HMV-Kp strains were positive for rmpA/rmpA2. MLST and K serotyping revealed that two isolates belong to ST23-K1 and ST86-K2, a known hypervirulent clone. All strains contained virulence genes, with the two ST23-K1 strains carrying all the typical hvKp virulence genes. No strains harbored ESBL or carbapenemase genes.

**Table 1.**
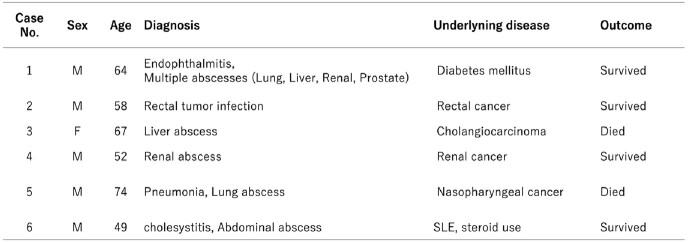
Clinical backgrounds of the cases.

**Table 2.**
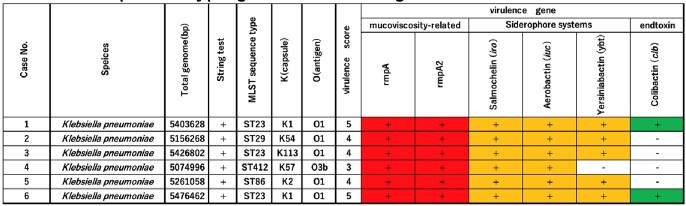
Sequence typing and Virulence genes of the isolates.

**Conclusion:**

All HMV-Kp strains isolated in this study carried virulent genes, including the hvKP clones ST23-K1 and ST86-K2. The String test for HMV phenotype confirmation appears useful to estimate the presence of hvKp. Our findings provide insights into the virulence and potential clinical implications of HMV-Kp isolates in Japan, highlighting the need for further investigation and monitoring.

**Disclosures:**

**Kazuko Yamamoto, MD, PhD**, Fisher & Paykel Healthcare: Grant/Research Support|Kirin Holdings Co.: Grant/Research Support

